# LncRNA HOXC-AS3 promotes non-small-cell lung cancer growth and metastasis through upregulation of YBX1

**DOI:** 10.1038/s41419-022-04723-x

**Published:** 2022-04-06

**Authors:** HongBo Su, GuanZhi Fan, Jin Huang, XueShan Qiu

**Affiliations:** 1 The First Affiliated Hospital and Basic Medical Sciences College of China Medical University, Shenyang, 110001 Liaoning Province P. R. China; 2 Department of Pathology, Shengjing Affiliated Hospital, China Medical University, Shenyang, 110001 Liaoning Province P. R. China; 3 Department of Radiotherapy, First Affiliated Hospital, China Medical University, Shenyang, 110001 Liaoning Province P. R. China

**Keywords:** Cell growth, Cancer

## Abstract

NSCLC is common and is the primary cause of cancer-related deaths due to a lack of early diagnosis and its propensity for metastasis. The pathogenesis of NSCLC is still unclear. Here, we explored the molecular mechanisms underlying NSCLC development, focusing on the HOXC-AS3/YBX1/HOXC8 axis. Human NSCLC specimens and cell lines were used. qRT-PCR and western blotting were utilised to examine the levels of HOXC-AS3/YBX1/HOXC8. CCK-8, colony formation, scratch wound healing and Transwell assays were performed to evaluate cancer cell proliferation, migration and invasion. A nude mouse xenograft model was used to examine tumour growth and metastasis in vivo. RNA pull-down, chromatin immunoprecipitation, coimmunoprecipitation and dual-luciferase assays were applied to validate the interactions of HOXC-AS3/YBX1, MDM2/YBX1 and the YBX1/HOXC8 promoter. The levels of HOXC-AS3 and HOXC8 were increased in human NSCLC specimens and cells. Knockdown of HOXC-AS3 suppressed NSCLC cell proliferation, migration and invasion, as well as tumour growth and metastasis in vivo. HOXC-AS3 directly bound to YBX1 to suppress its ubiquitination mediated by MDM2. YBX1 bound to the HOXC8 promoter and enhanced its transcription. Knockdown of HOXC8 inhibited the effects of HOXC-AS3 overexpression on NSCLC. HOXC-AS3 promotes NSCLC growth and metastasis by stabilising YBX1 and thus increasing HOXC8 transcription. Our study indicates that the HOXC-AS3/YBX1/HOXC8 axis could serve as a biomarker for NSCLC diagnosis or as a target for therapy development.

## Introduction

Lung cancer is the leading cause of cancer-related mortality around the world and it can be divided into two types: small cell lung cancer and non-small cell lung cancer (NSCLC) [1, 2]. The latter type accounts for ~80–90% of all lung cancer cases [1]. Often, the diagnosis of NSCLC is not made until advanced-stage symptoms are present, which makes treatment challenging. Patients in the early stages could be treated with surgical resection plus radiotherapy or chemotherapy [3, 4]. However, the treatment of patients in the advanced stages is limited, and the prognosis is very poor, primarily due to metastasis [3, 4]. Consequently, understanding the molecular mechanisms underlying the development and progression of NSCLC is critical to improve the outcomes.

The molecular biology of NSCLC is under intense scrutiny, and many molecules and signalling pathways have been implicated in NSCLC, such as immunocompromise- and hypoxia resilience-related molecules [5, 6]. In addition, numerous noncoding RNAs (ncRNAs), including long noncoding RNAs (lncRNAs), have been shown to significantly contribute to disease development and progression [7, 8]. For instance, lncRNA UFC1 promotes NSCLC cell proliferation, migration and invasion [9]. In contrast, lncRNA linc00961 suppresses the invasion and metastasis of NSCLC [10].

LncRNAs are a class of endogenous ncRNAs with lengths exceeding 200 nucleotides [11]. Accumulating evidence has shown that lncRNAs regulate many subcellular processes, such as cell proliferation and death, and thus are involved in the development of many diseases [11, 12]. Recently, a study reported an increase of lncRNA HOXC-AS3 in lung cancer cells [13]. Nevertheless, the exact functional role of lncRNA HOXC-AS3 in NSCLC and the underlying molecular mechanisms remain largely unknown.

LncRNA HOXC-AS3 is a natural antisense transcript of the HOXC10 gene that has been implicated in multiple cancers [14]. HOXC-AS3 has been shown to promote the progression of breast cancer, gastric cancer and invasive mucinous adenocarcinoma of the lung [13, 15, 16]. Although lncRNAs are not directly translated into proteins, they can exert their functions by binding to microRNAs or proteins, including transcriptional regulators [11]. HOXC-AS3 has been shown to interact with Y-box binding protein-1 (YBX1), a transcription factor, in gastric cancer cells [16], and our bioinformatic analysis (RNAInter) identified this interaction in NSCLC cells as well. Moreover, YBX1 has been reported to be upregulated in NSCLC cells [17]. Thus, we wondered whether HOXC-AS3 regulated NSCLC by binding to YBX1. In addition, in our preliminary analysis (hTFtarget), we identified some binding sites between YBX1 and the HOXC8 (homeobox C8) promoter region, suggesting that YBX1 could potentially regulate HOXC8 transcription. HOXC8 attracted our attention because it has also been implicated in NSCLC [18]. In view of the evidence presented above, we hypothesised that the HOXC-AS3/YBX1/HOXC8 axis might play a key role in NSCLC development.

In this study, we sought to fully elucidate the functional role of HOXC-AS3/YBX1/HOXC8 in NSCLC. We observed robust increases in HOXC-AS3/YBX1/HOXC8 levels in human NSCLC specimens and cells. Knockdown of HOXC-AS3 strongly suppressed NSCLC cell proliferation, migration and invasion, and it inhibited tumour growth and metastasis in vivo, while overexpression further facilitated NSCLC cell proliferation, migration and invasion. Notably, knockdown of HOXC8 blocked the effects of HOXC-AS3 overexpression. Mechanistically, we demonstrated that HOXC-AS3 directly bound to YBX1 to stabilise its expression by suppressing MDM2-mediated ubiquitination, while YBX1 interacted with the HOXC8 promoter to enhance its transcription. Taken together, our study reveals an essential role of the HOXC-AS3/YBX1/HOXC8 axis in NSCLC progression, shedding light on the molecular mechanisms of the disease and providing avenues for the development of therapeutic strategies.

## Materials and methods

### Human NSCLC specimen

Human NSCLC specimens and nearby nontumorigenic specimens were collected from 30 diagnosed NSCLC patients without preoperative treatment during surgery at the First Affiliated Hospital of China Medical University. All collected specimens were snap-frozen in liquid nitrogen and then stored at −80 °C until use. This study was approved by the ethics committee of the First Affiliated Hospital of China Medical University.

### Cell culture and transfection

Four human NSCLC cell lines (H522, H460, H1299 and A549) and one human nontumorigenic lung epithelial cell line (BEAS-2B) were utilised for this study and were purchased from the Cell Bank of the Chinese Academy of Sciences (Shanghai, China). All of the cells were cultured and maintained in DMEM (Gibco, China) with 10% foetal bovine serum (FBS, Gibco, China) and 1% antibiotics (penicillin–streptomycin). The cells were cultured in a CO_2_ incubator at 37 °C.

The full lengths of HOXC-AS3 and YBX1 were subcloned into the overexpression plasmid. Sh-HOXC-AS3, sh-HOXC8, sh-MDM2, sh-SYVN1 and control sh-NC were synthesised by GenePharma (Shanghai, China). Lipofectamine 3000 (Invitrogen, USA) was used for cell transfection according to the manufacturer’s instructions. Briefly, the cells were cultured to ~80% confluence. The corresponding constructs together with Lipofectamine 3000 (m/v 1:1) were directly added to the culture media for 48 h followed by harvest.

### Colony formation assay

NSCLC cells (2 × 103) were seeded into the wells of 12-well culture plates and grown for up to 2 weeks in a cell incubator. The cell medium was discarded, and 4% PFA was added to fix the cells and colonies at room temperature (RT) for 12-15 min followed by PBS washes. Crystal violet (0.1%) and methanol (20%) were added to stain the colonies. ImageJ software was used to quantify the number of colonies.

### Cell counting kit-8 (CCK-8)

The cell proliferation rate was measured by using the CCK-8 assay according to the manufacturer’s instructions. A total of 1 × 10^3^ cells were seeded into the wells of 96-well plates and grown in a cell incubator. At specific time points as indicated, CCK-8 solution (10 μL, Abcam, UK) was added and incubated with the cultured cells in the incubator for another 2 h followed by absorbance (490 nm) analysis with a microplate reader (Thomas Scientific, NJ, USA).

### Wound healing assay

Transfected NSCLC cells were seeded and cultured in six-well plates for 24 h until 90~95% confluence. A 10 μL pipette tip was used to make a straight line across the cell monolayer. The cell debris was washed off with PBS, and fresh media was added. The cells were put back in the incubator for 24 h. Images were taken at 0 h/24 h following wounding by a light microscope (Nikon, Japan). Image-Pro Plus software (Media Cybernetics, Inc., USA) was used to analyse the migration distances.

### Transwell invasion assay

To evaluate the invasion ability, transfected NSCLC cells or normal hepatic cells were seeded on a filter membrane (8 μm) precoated with Matrigel (Corning, NY, USA) in serum-free culture medium. Complete medium with 10% FBS was placed into the lower chamber. One day later, the filter was removed. Cells residing in the lower chamber had invaded the filter membrane, and they were first fixed in 4% PFA and subsequently stained with 0.1% crystal violet, followed by imaging.

### Dual-luciferase reporter assay

The wild–type (WT) or mutated (Mut) sequences with binding sites for YBX1 in the HOXC8 promoter regions (BS1-4) (WT-BS1:TCTTCCATC; WT-BS2: AGCCCCACC; WT-BS3: TGTTCCTCC; WT-BS4: CTCACCACC; Mut-BS1: AGAAGGTAG; Mut-BS2: TCGGGGTGG; Mut-BS3: ACAAGGAGG; Mut-BS4: GAGTGGTGG) were inserted into the pmirGLO luciferase reporter vector (Promega, WI, USA) (pmirGLO-HOXC8-WT-BS1, pmirGLO-HOXC8-Mut-BS1, pmirGLO-HOXC8-WT-BS2, pmirGLO-HOXC8-Mut-BS2, pmirGLO-HOXC8-WT-BS3, pmirGLO-HOXC8-Mut-BS3, pmirGLO-HOXC8-WT-BS4, pmirGLO-HOXC8-Mut-BS4). Mutagenesis was performed with a commercial kit (The Phusion Mutagenesis kit, Thermo-Fisher Scientific). HK-2 cells were seeded in 24-well plates one night before cotransfection with the luciferase constructs and YBX1 or control plasmid by using Lipofectamine 3000. After 48 h, the cells were collected and lysed to quantify the relative luciferase activities.

### Nude mice xenograft experiments

The animal experiment was carefully reviewed, approved and carried out under the guidance of the Animal Care and Use Committee of the First Affiliated Hospital of China Medical University. Adult nude male mice (BALB/c, 8 weeks old) were obtained from SJA Laboratory Animal Co., Ltd. (Hunan, China). Control NSCLC cells or NSCLC cells stably transfected with the corresponding plasmids (control sh-NC group, sh-HOXC-AS3-1 group, sh-HOXC-AS3-2 group, shHOXC8 group, HOXC-AS3 group and HOXC-AS3 + shHOXC8 group) were unilaterally subaxillarily subcutaneously injected into twelve-week-old nude mice (1 × 10^7^ cells per mouse) to induce tumours. The mice were monitored every day to observe tumour growth for 30 days. Tumour length (*L*) and width (*W*) were measured and the tumour volume (*V*) was calculated by *V*(mm^3^) = 0.5 × (*W*)^2^ × (*L*). At the end of the experiments, the tumours from each mouse were weighed.

To evaluate lung metastases, control NSCLC cells or transfected NSCLC cells (2 × 10^6^) (control sh-NC group, sh-HOXC-AS3-1 group, sh-HOXC-AS3-2 group, shHOXC8 group, HOXC-AS3 group and HOXC-AS3 + shHOXC8 group) were tail-injected into the mice through the vein. Thirty days later, the animals were sacrificed to harvest the lung tissues for further experiments (H&E staining).

### H&E staining

Lung tissues were fixed overnight in 4% PFA buffer at 4 °C and subsequently embedded in optimal cutting temperature compound. The tissues were sliced into 20 μm sections and stained with haematoxylin and eosin (H&E) according to the manufacturer’s instructions.

### RNA extraction and qRT-PCR

DNaseI containing TRIzol (Invitrogen, China) was added to the tissues/cultured cells to extract the total RNA following the manufacturer’s instructions. cDNA was generated via reverse transcription using a commercial kit (cDNA synthesis kit, Thermo-Fisher, China). After obtaining the cDNA, qPCR was carried out with the SYBR Green Master Mix (Invitrogen, China). The relative expression levels of HOXC-AS3 and HOXC8 mRNA were normalised to U6 or GAPDH mRNA as internal controls. The 2^−ΔΔCt^ method was used to quantify the expression levels. The primers were obtained from Guangzhou RiboBio Co., Ltd. (Guangdong, China):

HOXC-AS3 forward primer (F): 5′-TTGCGTGACAGTTTCCACTC-3′;

HOXC-AS3 reverse primer (R): 5′-CTCCCTTCTGCGGTCATTTC-3′;

HOXC8 mRNA F: 5′-CACGTCCAAGACTTCTTCCACC-3′;

HOXC8 mRNA R: 5′-CACTTCATCCTTCGATTCTGG-3′;

GAPDH F: 5′-CCAGGTGGTCTCCTCTGA-3′;

GAPDH R: 5′-GCTGTAGCCAAATCGTTGT-3′.

### RNA immunoprecipitation assay

Lysis buffer (50 mM Tris-HCl, 2.5 mM EDTA, 130 mM NaCl, 1% NP-40) supplemented with RNase and protease inhibitors (Thermo Scientific, China) was used to lyse the transfected cells. The protein concentration was determined, and equal amounts of protein samples were added and incubated with primary antibodies (anti-YBX1 or IgG as a control) (Thermo Fisher, USA) at 4 °C overnight. The antibody-conjugated samples were then incubated with protein A Sepharose (Sigma-Aldrich, USA) for 2 h at 4 °C. The samples were washed with washing buffer and then incubated with proteinase K (Sangon, China) for 1.5 h. The elution was subjected to RNA extraction by TRIzol reagent (Invitrogen, USA) followed by qRT-PCR. The primers used for measuring the RNA yield were the same as the primers in the qRT-PCR section.

### Chromatin immunoprecipitation assay

A chromatin immunoprecipitation (ChIP) kit (Cell Signaling Technology, USA) was employed for ChIP analysis. Briefly, the proteins were cross-linked to the DNA by adding formaldehyde to the medium followed by PBS washing and harvest. The cell suspension was sonicated, and then the cell debris was pelleted by centrifugation. We collected the supernatant and quantified the DNA concentration and size followed by chromatin immunoprecipitation. Ten micrograms of anti-YBX1 or 1 µL of normal IgG was incubated with the samples for 1 h at 4 °C followed by protein A bead incubation overnight. The beads were washed with washing buffer and then eluted with elution buffer followed by purification of chromosomal DNA. The HOXC8 promoter region was analysed by qRT-PCR..The ChIP primers are listed in as followed: HOXC8 BS1 F: 5′-ATGCCAGAGTTCTGGGTGTC-3′; HOXC8 BS1 R: 5′-TCCCAAAGGAGATTCACTGG-3′; HOXC8 BS2 F: 5′- CTCAGAGCCCCTAGCCAAG-3′; HOXC8 BS2 R: 5′-AGGAGAGGTCAGAGGAGGT -3′; HOXC8 BS3 F: 5′-ATGGTGTGCTTGTTCCTCCT-3′; HOXC8 BS3 R: 5′- GGTCAACCCAGGTTACCTCA-3′; HOXC8 BS4 F: 5′-TTCGTACCCTGGAAAACTG G-3′; HOXC8 BS4 R: 5′-GGGGTTGAGGGGTACAGG -3′;

### Biotinylated RNA pull-down assay

Transfected NSCLC cells were homogenised in lysis buffer, and the cell lysates were incubated with biotinylated HOXC-AS3 (generated with the tMEGshortscript^TM^ T7 kit) for 2 h at 4 °C. Streptavidin-coupled Dynabeads (Invitrogen, Shanghai, China) were added and incubated with the samples for an additional 3 h at 4 °C. The samples were washed with washing buffer, and the beads were then eluted with Laemmli buffer. The elution was subjected to western blotting.

### Western blot analysis

Tissues or cultured cells were homogenised in RIPA lysis buffer (Abcam, China) to extract the proteins. The protein samples were then processed with a BCA assay kit (Bio-Rad, China) to measure the protein concentration. Equal protein concentrations were loaded for electrophoresis followed by transfer to PVDF membranes (Bio-Rad, China). The membranes were then blocked in PBS containing 3% BSA buffer for 0.5–1 h at RT for 1 h and subsequently incubated with primary antibodies diluted in blocking buffer overnight on a shaker at 4 °C. The next day, the primary antibodies were washed off with TBST and then incubated with the specific secondary antibodies for 2 h on a shaker at RT followed by TBST washing. The signals were detected with an ECL kit. The following antibodies were used in this study: anti-HOXC8 (1:1500; Abcam, UK), anti-YBX1 (1:1500; Abcam, UK), anti-MDM2 (1:2500; Abcam, UK), anti-GAPDH (1:3000; Abcam, UK) and anti-β-actin (1:4000; Santa Cruz, USA).

### Coimmunoprecipitation

Transfected NSCLC cells were lysed with lysis buffer (50 mM Tris-HCl, 200 mM NaCl, 1 mM EDTA, 1% Triton X-100) supplemented with protease inhibitor. Protein samples were processed with a BCA assay kit (Bio-Rad China) to measure the protein concentration. The same amounts of proteins were incubated with anti-YBX1 antibody (Abcam, UK), anti-MDM2 (Abcam, UK), or IgG antibody (Thermo Fisher, USA) overnight at 4 °C, and then Protein-A Dynabeads (Thermo Fisher) were added for an additional 3 h incubation at 4 °C. The beads were then washed in lysis buffer followed by elution with SDS loading buffer. The elution was subjected to standard western blotting.

### Statistical analysis

All experiments were performed with at least three biological replicates, and the experimental data were plotted in GraphPad Prism 7. Statistics were calculated by using unpaired Student’s *t* test (for two groups) or one-way ANOVA (for more than two groups), and the statistical details are indicated in the figure legends. Pearson correlation coefficient analysis was used to evaluate the relationship between HOXA3-AS and HOXC8 mRNA. The data are presented as the mean ± SD (standard deviation).

## Results

### HOXC-AS3 and HOXC8 were increased in NSCLC tissues and cells

**Fig. 1:**
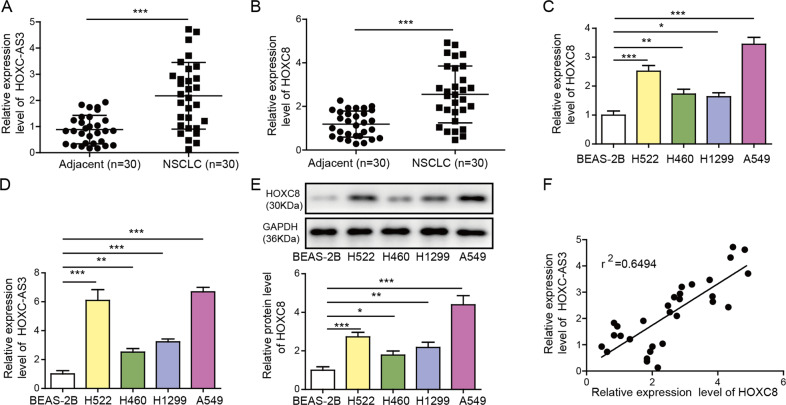
HOXC-AS3 and HOXC8 are elevated in NSCLC tissues and cells. **A** Relative HOXC-AS3 levels in NSCLC tissues and adjacent normal tissues from diagnosed human NSCLC patients. **B** Relative HOXC8 mRNA levels in NSCLC tissues and nearby adjacent normal tissues from diagnosed human NSCLC patients. **C** Relative HOXC-AS3 levels in NSCLC cell lines and nontumorigenic lung epithelial cells. **D** Relative HOXC8 mRNA levels in NSCLC cell lines and nontumorigenic lung epithelial cells. **E** Relative HOXC8 protein levels in NSCLC cell lines and nontumorigenic lung epithelial cells. **F** Correlation between HOXC-AS3 levels and HOXC8 mRNA levels in NSCLC tissues from human patients. *p < 0.05, **p < 0.01, ***p < 0.001.

To investigate the functions of HOXC-AS3 and HOXC8 in NSCLC, we first measured their expression levels in NSCLC cells. We obtained NSCLC tissues and nearby non-tumour tissues from diagnosed NSCLC patients receiving no preoperative treatments. First, we observed that both HOXC-AS3 and HOXC8 were elevated in NSCLC tissues compared to adjacent noncancer tissues (Fig. 1A, B). To further study their function, we utilised four NSCLC cell lines. Compared to nontumorigenic lung epithelial cells, the levels of HOXC-AS3 and HOXC8 mRNA were remarkably upregulated in NSCLC cells (Fig. 1C, D). Consistently, HOXC8 protein levels were increased in NSCLC cells (Fig. 1E). Moreover, we detected a very strong positive correlation between HOXC-AS3 levels and HOXC8 mRNA levels in human NSCLC tissues (Fig. 1F). Altogether, these data show that HOXC-AS3 and HOXC8 are upregulated in NSCLC cells.

### Knockdown of HOXC-AS3 suppressed NSCLC cell proliferation, migration and invasion

**Fig. 2:**
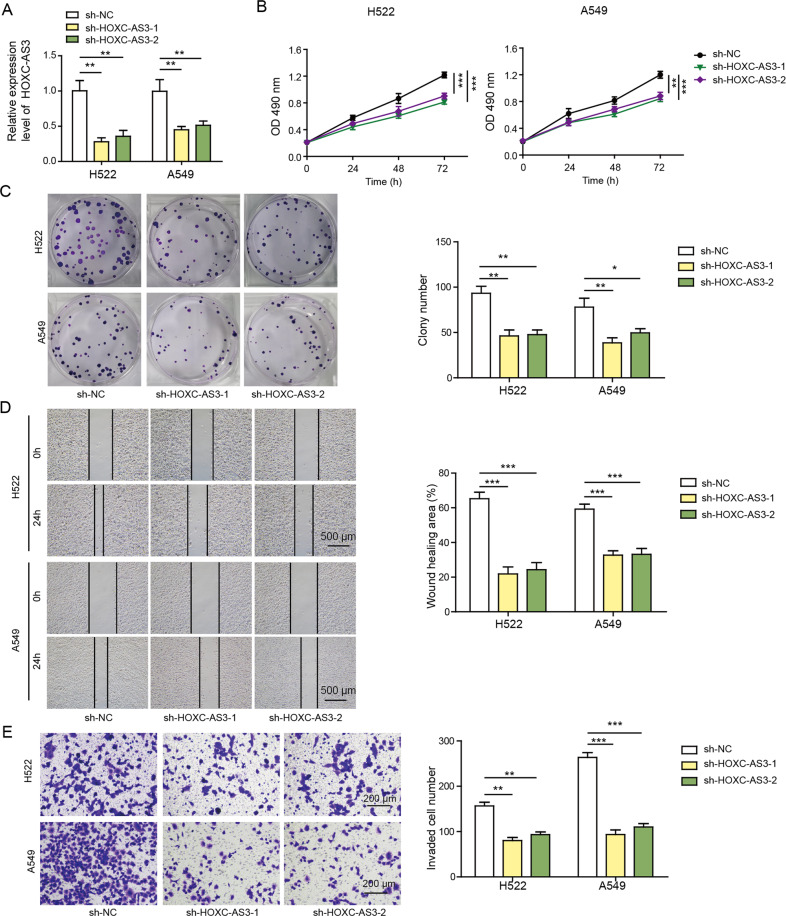
Knockdown of HOXC-AS3 suppressed NSCLC cell proliferation, migration and invasion. **A** Relative HOXC-AS3 levels in NSCLC cells transfected with sh-NC or sh-HOXC-AS3. **B** CCK-8 assay to assess the proliferation rate of transfected NSCLC cells. **C** Colony formation assay to analyse the proliferation rate of transfected NSCLC cells. **D** Scratch wound healing assay to evaluate the migration ability of transfected NSCLC cells. **E** Transwell assay to quantify the number of invasive cells following transfection of sh-NC or sh-HOXC-AS3. *p < 0.05, **p < 0.01, ***p < 0.001.

Next, we examined how HOXC-AS3 regulated NSCLC. Since the levels of HOXC-AS3 and HOXC8 were higher in H522 and A549 cells than in H460 and H1299 cells (Fig. 1C–E), we used H522 and A549 cell lines for subsequent experiments. As expected, transfection of cells with sh-HOXC-AS3 dramatically reduced its level (Fig. 2A). With a CCK-8 assay, we found that knockdown of HOXC-AS3 significantly suppressed NSCLC cell proliferation (Fig. 2B). Similarly, the colony formation assay results showed that cells transfected with sh-HOXC-AS3 formed fewer colonies than sh-NC-transfected cells (Fig. 2C). Furthermore, with a scratch wound healing assay, we observed that the migration distance of cells with HOXC-AS3 knockdown for 24 h was smaller than that of cells transfected with sh-NC (Fig. 2D). In addition, the Transwell assay data indicated that knockdown of HOXC-AS3 significantly decreased the number of invasive cells (Fig. 2E). Taken together, these results demonstrate that knockdown of HOXC-AS3 suppresses NSCLC cell proliferation, migration and invasion.

### Knockdown of HOXC-AS3 inhibited NSCLC tumour growth and metastasis in vivo

**Fig. 3:**
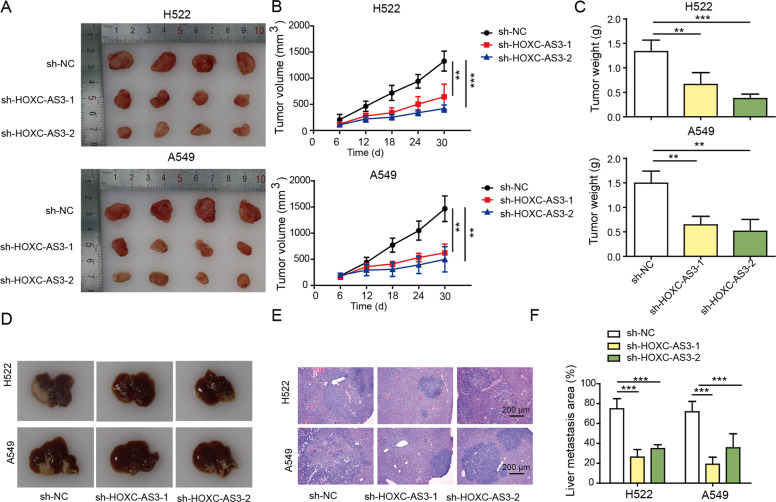
Knockdown of HOXC-AS3 inhibited NSCLC tumour growth and metastasis in vivo. **A** Representative images of tumours from individual groups. **B** Quantification of the tumour volume from individual groups at the indicated time points. **C** Quantification of the tumour weight from individual groups at Day 30. **D** Representative images of tumour nodules in the liver of individual groups. **E** Representative H&E staining images of the liver in each group. **F** Quantification of the liver metastasis area in the liver of each group. **p < 0.01, ***p < 0.001.

We further studied how HOXC-AS3 regulated NSCLC tumour growth and invasion in vivo by using a nude mouse xenograft model. We injected nude mice with sh-NC-transfected or sh-HOXC-AS3-transfected H522 and A549 cells. As shown in Fig. 3A, B, the tumours in mice injected with shNC-transfected NSCLC cells grew rapidly, and the tumour volume progressively increased. However, the tumour volume in the sh-HOXC-AS3 groups was consistently and significantly smaller than that in the sh-NC group (Fig. 3A, B). In the end, the tumour weight in the sh-HOXC-AS3 groups was significantly reduced in comparison to that in the sh-NC group (Fig. 3C). To evaluate tumour metastasis, we injected NSCLC cells into the tail vein and examined tumour growth in the liver. As expected, we observed many tumour nodules in the liver of the sh-NC group (Fig. 3D–F). In contrast, the metastatic area in the liver of the sh-HOXC-AS3 group was significantly smaller than that in the sh-NC group (Fig. 3D–F). Therefore, we concluded that knockdown of HOXC-AS3 inhibits NSCLC tumour growth and metastasis in vivo.

### HOXC-AS3 directly bound to YBX1 to decrease its ubiquitination

**Fig. 4:**
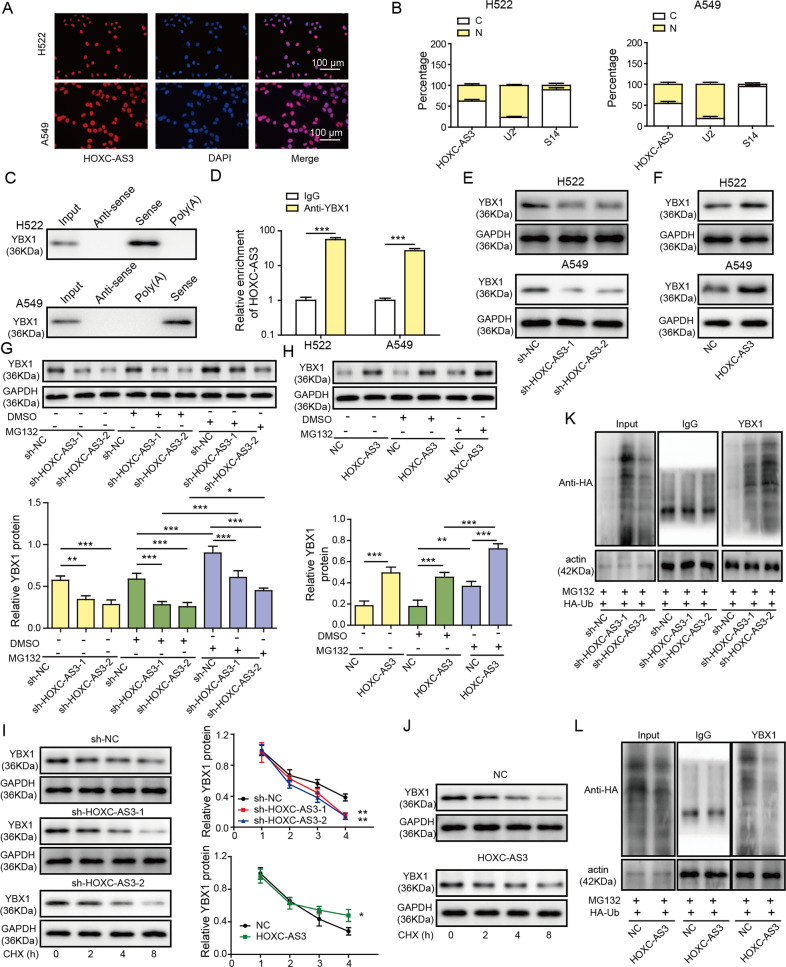
HOXC-AS3 is directly bound with YBX1 to decrease its ubiquitination. **A** FISH analysis of the subcellular localisation of HOXC-AS3. **B** Relative abundance of HOXC-AS3, U2 and S14 in the cytoplasm and nucleus. **C** Immunoblotting of YBX1 following immunoprecipitation with specific RNA probes. **D** Relative HOXC-AS3 levels following immunoprecipitation with YBX antibody or IgG antibody. **E** Relative YBX1 protein levels in cells transfected with shNC or sh-HOXC-AS3. **F** Relative YBX1 protein levels in cells transfected with NC or OE-HOXC-AS3. **G** Relative YBX1 protein levels in cells transfected with shNC or sh-HOXC-AS3 with or without MG132 treatment. **H** Relative YBX1 protein levels in cells transfected with NC or OE-HOXC-AS3 with or without MG132 treatment. **I** Relative YBX1 protein levels in cells transfected with shNC or sh-HOXC-AS3 following CHX treatment. **J** Relative YBX1 protein levels in cells transfected with shNC or sh-HOXC-AS3 following CHX treatment. **K** Immunoblotting for HA following immunoprecipitation with YBX1 antibody in cells transfected with HA-Ub together with shNC or sh-HOXC-AS3 and treated with MG132. **L** Immunoblotting for HA following immunoprecipitation with YBX1 antibody in cells transfected with HA-Ub together with NC or OE-HOXC-AS3 and treated with MG132. ***p < 0.001.

Next, we sought to investigate the molecular mechanisms underlying the function of HOXC-AS3 in NSCLC. It has been shown that lncRNAs regulate subcellular processes by sponging miRNAs or directly binding to proteins [11]. Through bioinformatic analysis (RNAInter), we found that HOXC-AS3 might potentially interact with YBX1, a transcription factor that has been implicated in NSCLC. We thus wondered whether HOXC-AS3 might regulate NSCLC through YBX1. With subcellular fractionation and FISH, we first observed that HOXC-AS3 was expressed mainly in the nucleus of NSCLC cells (A549) (Fig. 4A, B). With an RNA pulldown assay, we found that the HOXC-AS3 sense strand pulled down significantly more YBX1 protein in A549 cells (Fig. 4C). On the other hand, the YBX1 antibody pulled down more HOXC-AS3 than IgG (Fig. 4D). These results demonstrate that HOXC-AS3 directly binds to YBX1 in the nucleus.

We examined whether HOXC-AS3 regulated YBX1 expression. The western blotting results showed that knockdown of HOXC-AS3 with sh-HOXC-AS3 significantly diminished YBX1 protein expression, while overexpression of HOXC-AS3 upregulated YBX1 levels in A549 cells (Fig. 4E, F). To study the underlying mechanisms, we performed bioinformatic analysis with ubibrowser and found that two E3 ubiquitin-protein ligases, namely, MDM2 (mouse double minute 2 homologue) and SYVN1 (E3 ubiquitin-protein ligase synoviolin), could potentially promote the ubiquitination and proteasomal degradation of YBX1. We directly examined whether they modulated YBX1 ubiquitination. Knockdown of MDM2 strongly decreased the ubiquitination level of YBX1, while knockdown of SYVN1 did not (Fig. S1), suggesting that MDM2 but not SYVN1 mediates the ubiquitination of YBX1. Therefore, we focused on MDM2 for subsequent investigation. We examined whether HOXC-AS3 regulated YBX1 expression by modulating the ubiquitination process. Notably, we observed that MG132, a proteasome inhibitor, increased the YBX1 protein levels in A549 cells (Fig. 4G). Moreover, treatment with MG132 significantly suppressed the reduction in YBX1 levels caused by sh-HOXC-AS3 (Fig. 4G). Additionally, treatment with MG132 further increased YBX1 levels in OE-HOXC-AS3-transfected cells compared with OE-HOXC-AS3-transfected cells alone (Fig. 4H). In contrast, treatment with CHX, a protein synthesis inhibitor, gradually decreased YBX1 levels over time (Fig. 4I). Furthermore, CHX accelerated the reduction in YBX1 levels in sh-HOXC-AS3-transfected cells (Fig. 4I). In cells overexpressing HOXC-AS3, the decrease in YBX1 levels caused by CHX treatment was mitigated (Fig. 4J). Finally, we directly measured how HOXC-AS3 modulated the ubiquitination of YBX1. We overexpressed HA-Ub in A549 cells. As shown in Fig. 4K, ubiquitinated YBX1 was significantly increased in cells transfected with sh-HOXC-AS3 as compared to sh-NC transfected cells. In contrast, the ubiquitination level of YBX1 was significantly decreased in cells overexpressing HOXC-AS3 (Fig. 4L). Taken together, these results provide evidence that HOXC3-AS3 directly binds with YBX1 and stabilises YBX1 levels by suppressing the ubiquitination and degradation of YBX1.

### HOXC-AS3 disrupted the binding of MDM2 with YBX1

**Fig. 5:**
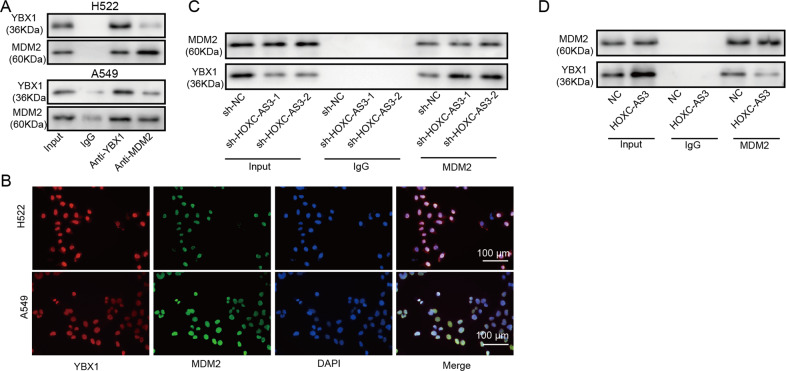
HOXC-AS3 disrupted the binding of MDM2 with YBX1. **A** Immunoblotting for YBX1 and MDM2 following immunoprecipitation with an MDM2 antibody. **B** Immunostaining analysis of YBX1 and MDM2 in NSCLC cells. **C** Immunoblotting for YBX1 and MDM2 following immunoprecipitation with MDM2 antibody in cells transfected with shNC or sh-HOXC-AS3. **D** Immunoblotting for YBX1 and MDM2 following immunoprecipitation with MDM2 antibody in cells transfected with NC or OE-HOXC-AS3.

We next investigated the molecular mechanisms of how HOXC-AS3 regulated the ubiquitination of YBX1. Based on our bioinformatic analysis, we found that MDM2 might regulate the ubiquitination and proteasomal degradation of YBX1. To validate this, we first performed a coimmunoprecipitation experiment and confirmed that MDM2 directly interacted with YBX1, as the MDM2 antibody pulled down significantly more YBX1 than the IgG antibody (Fig. 5A). Next, we performed immunostaining and observed that MDM2 colocalized with YBX1 in the nucleus (Fig. 5B). We then wondered whether HOXC-AS3 affected the binding between MDM2 and YBX1. As shown in Fig. 5C, the MDM2 antibody pulled down more YBX1 in cells transfected with sh-HOXC-AS3 than in sh-NC-transfected cells (Fig. 5C). In contrast, less YBX1 was pulled down in cells overexpressing HOXC-AS3 by the MDM2 antibody (Fig. 5D). These data show that HOXC-AS3 disrupts the interaction of MDM2 with YBX1.

### HOXC-AS3 enhanced NSCLC cell proliferation, migration and invasion induced by HOXC8

**Fig. 6:**
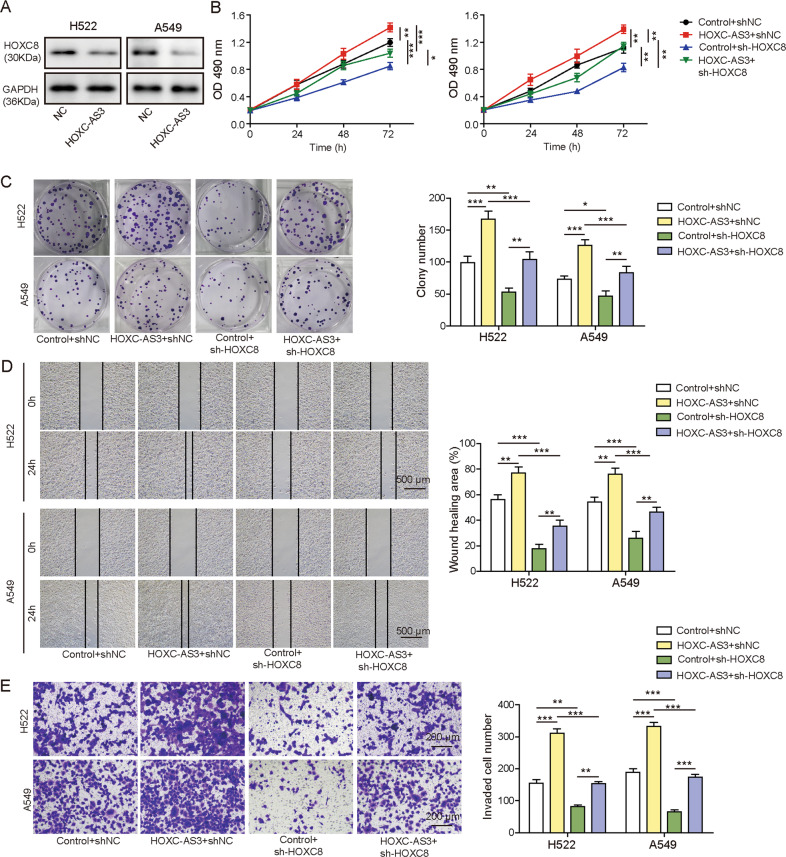
HOXC-AS3 enhanced NSCLC cell proliferation, migration and invasion by HOXC8. **A** Relative HOXC8 levels in cells transfected with NC or OE-HOXC-AS3. **B** CCK-8 assay to examine the proliferation rate of transfected cells. **C** Colony formation assay to examine the proliferation rate of transfected cells. **D** Scratch wound healing assay to evaluate the migration capacity of transfected cells. **E** Transwell assay to examine the invasion ability of transfected cells. *p < 0.05, **p < 0.01, ***p < 0.001.

HOXC8 has been shown to promote NSCLC cell proliferation, migration and invasion [18, 19]. We wondered how HOXC-AS3 affected this regulation. Overexpression of HOXC-AS3 significantly upregulated HOXC8 protein levels in NSCLC cells (Fig. 6A). With a CCK-8 assay, we found that overexpression of HOXC-AS3 increased the proliferation rate of NSCLC cells, while knockdown of HOXC8 decreased the proliferation rate (Fig. 6B). Moreover, knockdown of HOXC8 suppressed the increase in cell proliferation induced by HOXC-AS3 overexpression (Fig. 6B). Similarly, the colony formation assay results indicated that knockdown of HOXC8 significantly reduced the number of colonies formed and further inhibited the increase caused by HOXC-AS3 overexpression (Fig. 6C). Regarding migration, we showed that the migration distance was diminished in cells transfected with sh-HOXC8 (Fig. 6D). Again, the distance of cells cotransfected with sh-HOXC8 and HOXC-AS3 was reduced compared with that of cells transfected with HOXC-AS3 (Fig. 6D). The Transwell assay results showed that sh-HOXC8 significantly diminished the invasion of NSCLC cells and suppressed the increase in invasion mediated by HOXC-AS3 overexpression (Fig. 6E). These results show that HOXC-AS3 promotes NSCLC cell proliferation, migration and invasion via HOXC8.

We next examined the function of the HOXC-AS3/HOXC8 interaction in NSCLC in vivo. As shown in Fig. S2A–C, knockdown of HOXC8 robustly suppressed tumour growth with significantly reduced tumour volume and weight compared to the control group. Overexpression of HOXC-AS3 increased the tumour volume and weight (Fig. S2A–C). Nevertheless, co-overexpression of sh-HOXC8 together with HOXC-AS3 suppressed the increase in tumour volume and weight caused by HOXC-AS3 overexpression (Fig. S2A–C). Similarly, regarding tumour metastasis, we found that knockdown of HOXC8 greatly decreased the metastatic area of tumours in the liver compared to the control group, while overexpression of HOXC-AS3 increased the metastatic area (Fig. S2D–E). Again, sh-HOXC8 inhibited the increase mediated by HOXC-AS3 overexpression (Fig. S2D–E). Therefore, we conclude that HOXC-AS3 promotes NSCLC tumour growth and metastasis in vivo by increasing HOXC8 expression.

### HOXC-AS3 enhanced HOXC8 expression by increasing the binding of its promoter with YBX1

**Fig. 7:**
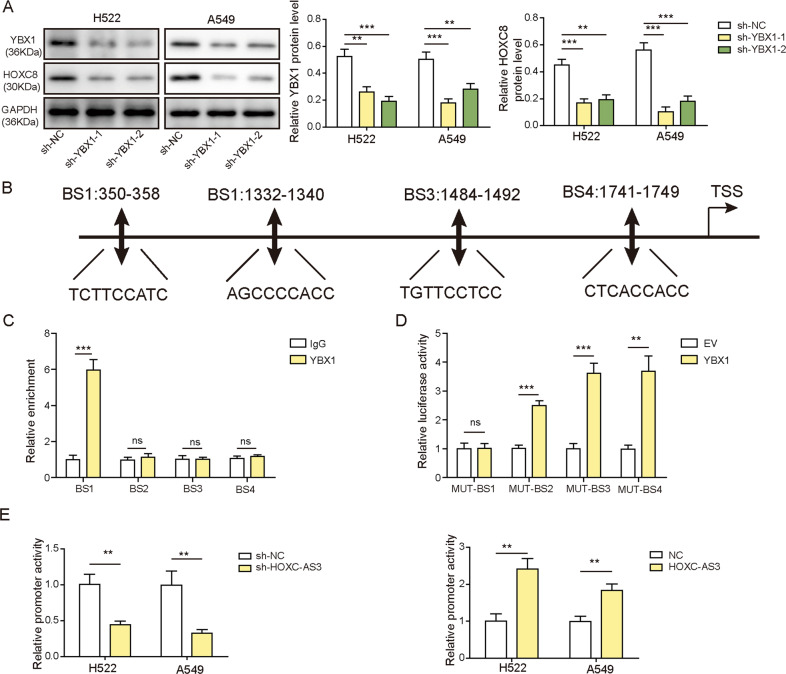
HOXC-AS3 enhanced HOXC8 expression by increasing the binding of its promoter with YBX1. **A** Relative HOXC8 levels in cells transfected with sh-NC or sh-YBX1. **B** The predicted binding sites between YBX1 and the HOXC8 promoter region. **C** Relative levels of BS1-4 following immunoprecipitation with YBX1 antibody or IgG antibody. **D** Relative luciferase activities of WT-BS1-4 and Mut-BS1 in cells transfected with YBX1. **E** Relative luciferase activities of the HOXC8 promoter in cells transfected with sh-HOXC-AS3 or OE-HOXC-AS3. *p < 0.05, **p < 0.01, ***p < 0.001, ns not significant.

Finally, we studied the molecular mechanisms by which HOXC-AS3 regulates NSCLC via HOXC8. As mentioned before, YBX1 is a transcription factor, and we have shown that HOXC-AS3 stabilises YBX1 by disrupting its interaction with MDM2. In addition, our aforementioned results indicated that overexpression of HOXC-AS3 upregulated HOXC8 levels (Fig. 6A). We thus hypothesised that HOXC-AS3 regulated HOXC8 expression via YBX1. To test this hypothesis, we first examined whether YBX1 regulated HOXC8 expression. The western blotting results showed that knockdown of YBX1 through shRNAs greatly diminished HOXC8 protein levels in NSCLC cells (Fig. 7A). Next, through bioinformatic analysis (JASPAER), we identified multiple binding sites between YBX1 and HOXC8 promoter regions (Fig. 7B). To confirm the binding, we selected 4 regions (BS1-4) with the highest potential (Fig. 7B) and performed ChIP experiments. As shown in Fig. 7C, we found that the YBX1 antibody pulled down significantly more BS1 than the IgG antibody but not BS2-4. Alternately, we employed a dual luciferase assay to directly examine the binding. We found that YBX1 greatly increased the luciferase activities of Mut-BS2, Mut-BS3 and Mut-BS4 but not Mut-BS1 (Fig. 7D). These results demonstrate that YBX1 directly binds to the HOXC8 promoter region. Next, we wondered how HOXC-AS3 affected the binding. We transfected NSCLC cells with sh-HOXC-AS3 or OE-HOXC-AS3 and then examined the ensuing effect on the luciferase activity of the HOXC8 promoter mediated by YBX1. Intriguingly, we observed that knockdown of HOXC-AS3 significantly decreased the luciferase activity of the HOXC8 promoter, while increasing HOXC-AS3 levels through overexpression greatly enhanced the activity (Fig. 7E). Taken together, these data show that YBX1 directly binds to the HOXC8 promoter to increase its expression and that HOXC-AS3 further strengthens the interaction and boosts the enhancement.

## Discussion

**Fig. 8:**
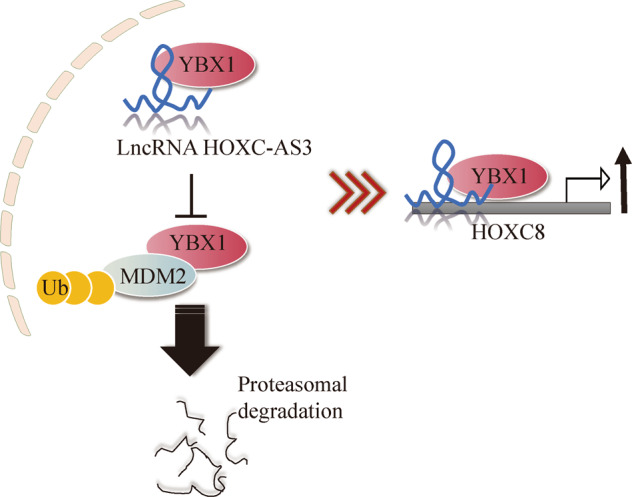
The model of the mechanism of HOXC-AS3 regulating HOXC8 in NSCLC tumour progression. Proposed model of the role of HOXC-AS3 to regulate HOXC8 for NSCLC tumour progression through competing with MDM2 to bind YBX1.

Owing to the difficulty of early diagnosis and high metastasis, the prognosis of NSCLC is poor [20, 21]. Currently, the molecular mechanism of NSCLC is still under scrutiny to search for new biomarkers for diagnosis or targets for treatment [6, 22, 23]. Here, we reported that HOXC-AS3/YBX1/HOXC8 plays an important role in NSCLC development and progression. Elevated expression of HOXC-AS3/YBX1/HOXC8 occurs in NSCLC cells, and inhibition of their expression significantly suppresses cancer cell proliferation, migration and invasion, resulting in attenuated tumour growth and metastasis in vivo. At the molecular level, we showed that HOXC-AS3 competed with MDM2 to bind YBX1 and stabilised its expression. Elevated YBX1 expression increased the transcription of HOXC8, as YBX1 bound to its promoter, leading to enhanced cancer cell proliferation, migration and invasion (Fig. 8). Altogether, these findings provide insights into the molecular mechanisms behind the progression of NSCLC.

HOXC-AS3 is a natural antisense transcript of the HOXC10 gene and it is encoded from chromosome 12q13.13 [14]. HOX genes are critical for development and morphogenesis, and aberrant expression of HOX genes is involved in many diseases, including cancers [24, 25]. As a result, lncRNA generation from HOX genes has a key role in physiological processes and in diseases. For instance, HOXC-AS3 has been shown to regulate mesenchymal stromal cell osteogenesis by interacting with HOXC10 [14]. In addition, HOXC-AS3 has been implicated in various types of cancers. HOXC-AS3 is upregulated in gastric cancer cells and promotes tumorigenesis [16]. In breast cancer, a high level of HOXC-AS3 is highly correlated with a poor prognosis [15, 26]. Here, we observed an increase in HOXC-AS3 levels in human NSCLC tissues and cells. Moreover, we showed that knockdown or overexpression of HOXC-AS3 significantly suppressed or enhanced cancer cell proliferation and invasion, as well as cancer growth and metastasis in vivo, respectively. Our study, together with previous research, indicates that HOXC-AS3 functions as a tumour oncogene in many types of cancers. It might be interesting to examine whether HOXC-AS3 has a similar tumour-promoting role in other cancers.

It is widely acknowledged that many lncRNAs are functional molecules, although they are not directly translated into functional proteins [11, 27]. A myriad of mechanisms has been reported through which lncRNAs regulate gene expression and thereby exert their functions [11]. Some lncRNAs directly interact with chromatin-modifying complexes to regulate chromatin structure [28]; some bind to the transcriptional machinery [29]; some lncRNAs act as competing endogenous RNAs for microRNAs [30]; others function posttranscriptionally as modulators of protein translation, stability, or mRNA decay [31]. In this study, we revealed that HOXC-AS3 regulates NSCLC progression by stabilising YBX1. We showed that HOXC-AS3 directly interacted with YBX1 and thus disrupted its binding to the E3-ubiquitin-protein ligase MDM2. As a result, the ubiquitination of YBX1 is suppressed. Further investigation is required to examine the exact type of ubiquitination modification on YBX1. Notably, HOXC-AS3 has been reported to act as a microRNA sponge [26]. It can bind to miR-3922-5p to promote breast cancer metastasis [26]. Therefore, HOXC-AS3 can exert functions through multiple mechanisms. Future studies are necessary to examine whether other mechanisms or binding partners of HOXC-AS3 are involved in NSCLC.

YBX1 is a well-known multifunctional oncoprotein that has been implicated in various types of cancers [32–34]. As a transcription factor, YBX1 regulates a wide range of genes that are involved in cell proliferation, growth, migration and drug resistance [32]. A large number of YBX1 targets have been revealed, such as growth factor receptor genes (*HER2/ErbB2, EGFR*), ATP binding cassette transporter genes (*ABCB1*) and drug resistance-associated genes (*MYC, MVP/LRP, CD44*) [34–36]. In this study, we identified the HOXC8 gene as a new target of YBX1 in NSCLC. We showed that YBX1 directly binds to the BS1 region of the HOXC8 promoter and activates HOXC8 expression. Moreover, we found that HOXC-AS3 strengthened the interaction and further enhanced this transcriptional regulation in NSCLC cells. Knockdown of HOXC8 reversed the promoting effects of HOXC-AS3 overexpression on NSCLC, indicating that YBX1/HOXC8 is a key downstream mechanism mediating the function of HOXC-AS3 in NSCLC. Whether HOXC-AS3 affects the binding of YBX1 with other genes remains to be further explored.

In conclusion, through a combination of in vitro assays and in vivo methods, we elucidated that HOXC-AS3 promotes NSCLC progression by stabilising YBX1 expression and thus enhancing HOXC8 transcription. The HOXC-AS3/YBX1/HOXC8 axis could serve as a biomarker for NSCLC diagnosis or as a therapeutic target to treat the disease.

## Supplementary information

supplemental Figure

WB

aj-checklist

Language Editing Certificate

## Data Availability

The datasets used or analyzed during the current study are available from the corresponding author on reasonable request.
