# Supplementary material for: LncRNA HOXC-AS3 promotes non-small-cell lung cancer growth and metastasis through upregulation of YBX1
Source: Cell Death Dis. 2022 Apr 6;13(4):307. doi: 10.1038/s41419-022-04723-x (PMC8986809; doi:10.1038/s41419-022-04723-x)
Supplement: Supplementary file 1 — supplemental Figure [file 41419_2022_4723_MOESM1_ESM.pdf]

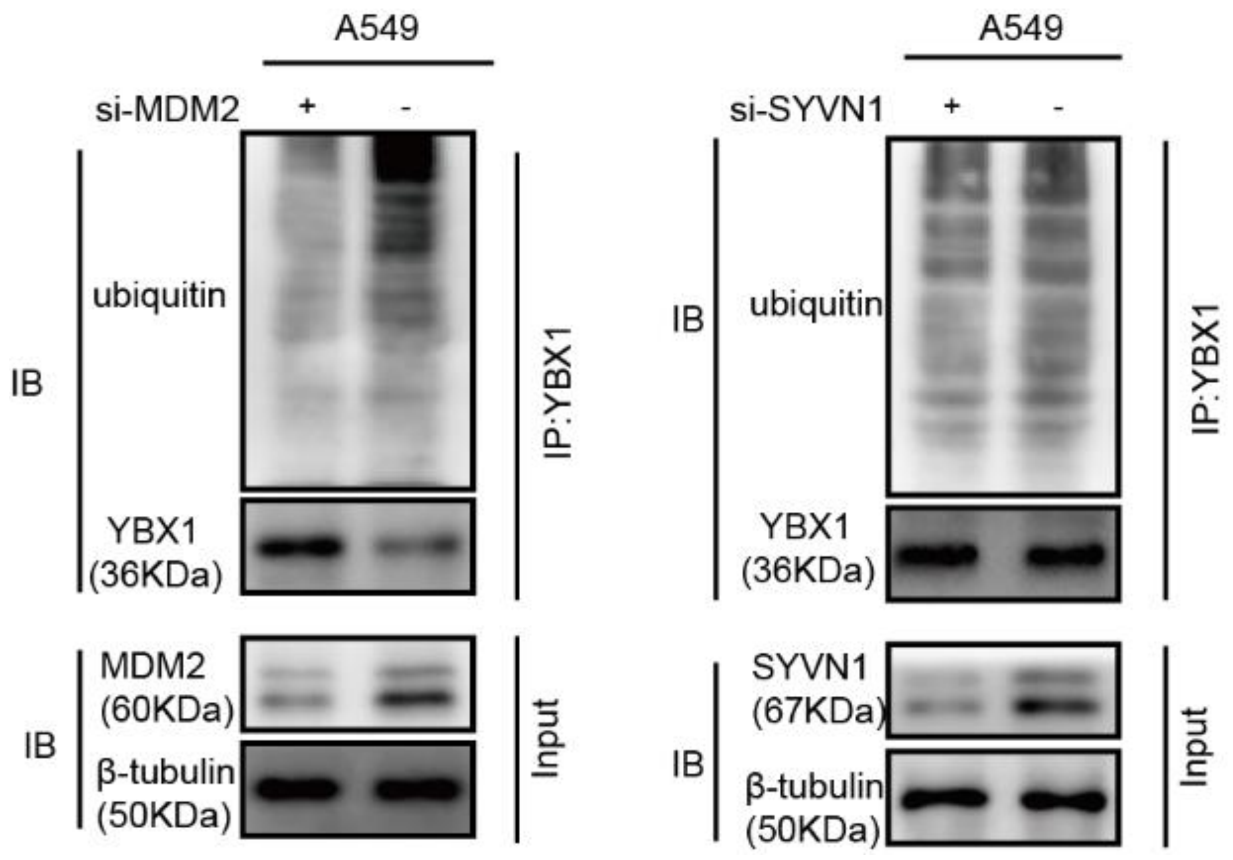

Fig.S1 Knockdown of MDM2 decreased the YBX1 ubiquitination level.
Immunoblotting for ubiquitination following YBX1 immunoprecipitation in A549 cells transfected with sh-NC, sh-MDM2, or sh-SYVN1.

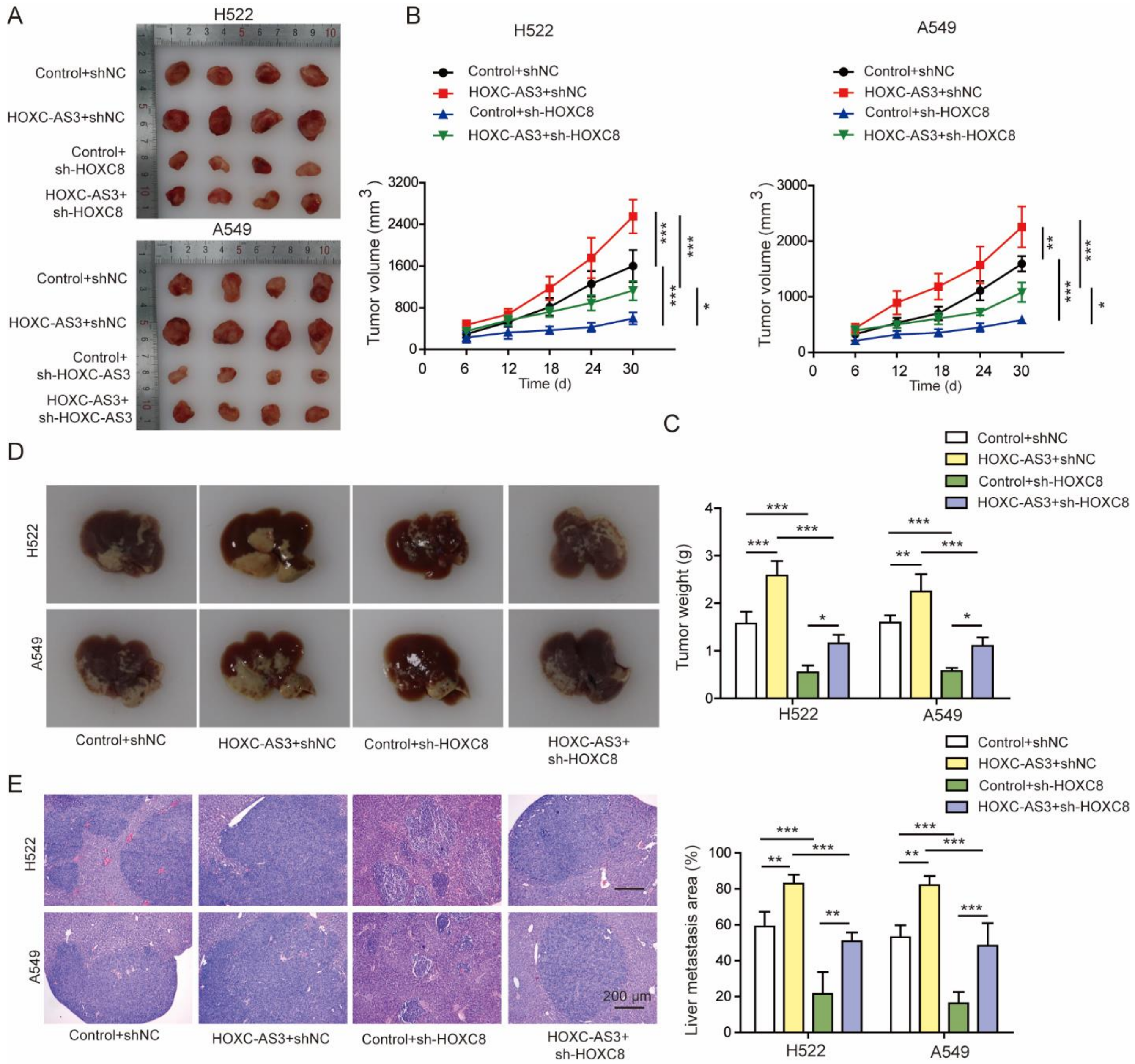

Fig.S2 HOXC-AS3 enhanced NSCLC cell proliferation and metastasis by HOXC8.

(A-C) Tumour volume and weight in each individual group. (A) Representative images of tumours from individual groups. (B) Quantification of the tumour volume from individual groups at the indicated time points. (C) Quantification of the tumour weight from individual groups at Day 30. (D-E) The number of tumour nodules in the liver of individual groups. (D) Representative images of tumour nodules in the livers of individual groups. (E) Representative H&E staining images of the liver in each group and quantification of the liver metastasis area in the liver of each group. $*p < 0.05$, $**p < 0.01$, $***p < 0.001$.
