# Supplementary figures and images for: LncRNA HOXC-AS3 promotes non-small-cell lung cancer growth and metastasis through upregulation of YBX1
Source: Cell Death Dis. 2022 Apr 6;13(4):307. doi: 10.1038/s41419-022-04723-x (PMC8986809; doi:10.1038/s41419-022-04723-x)

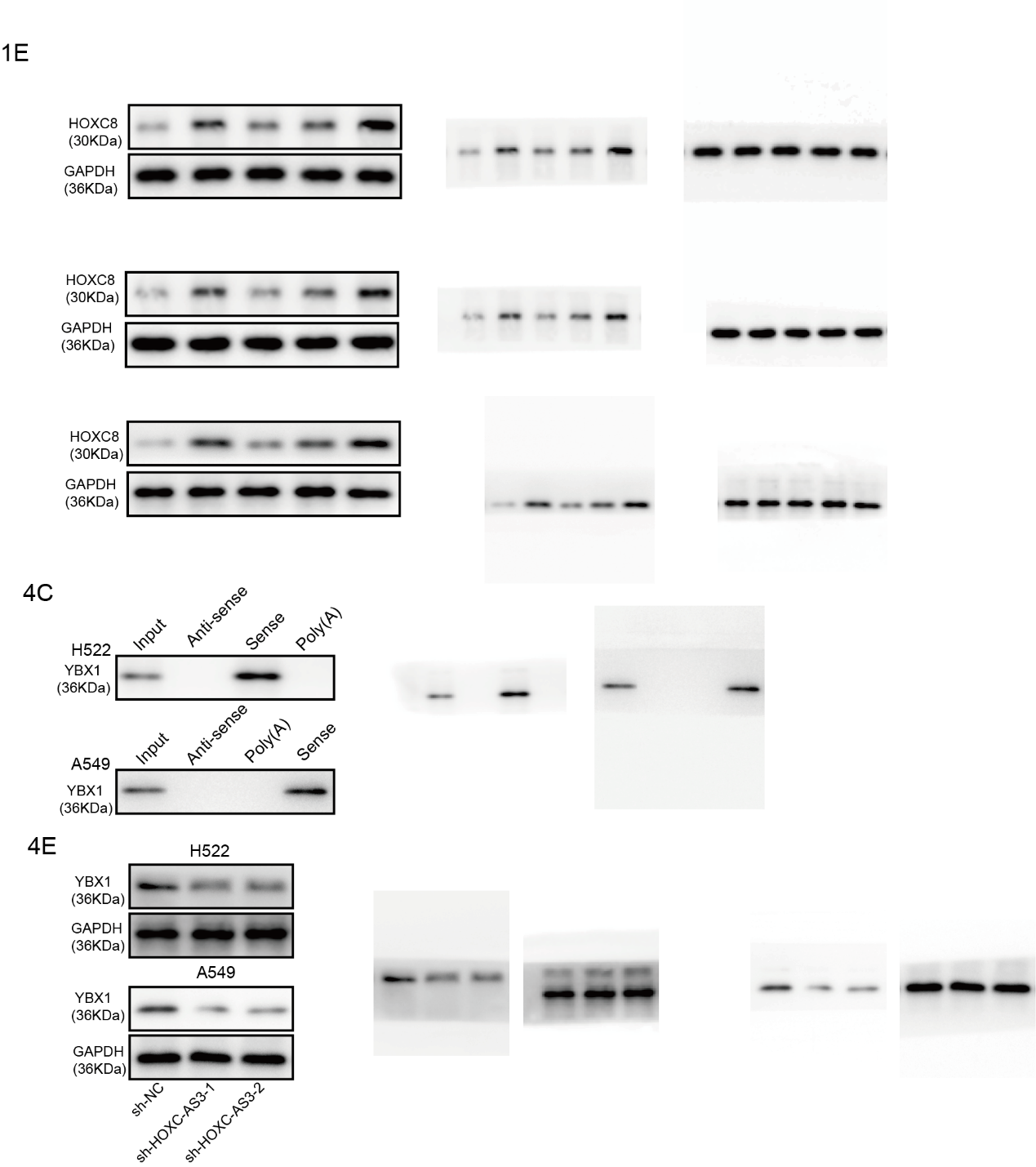

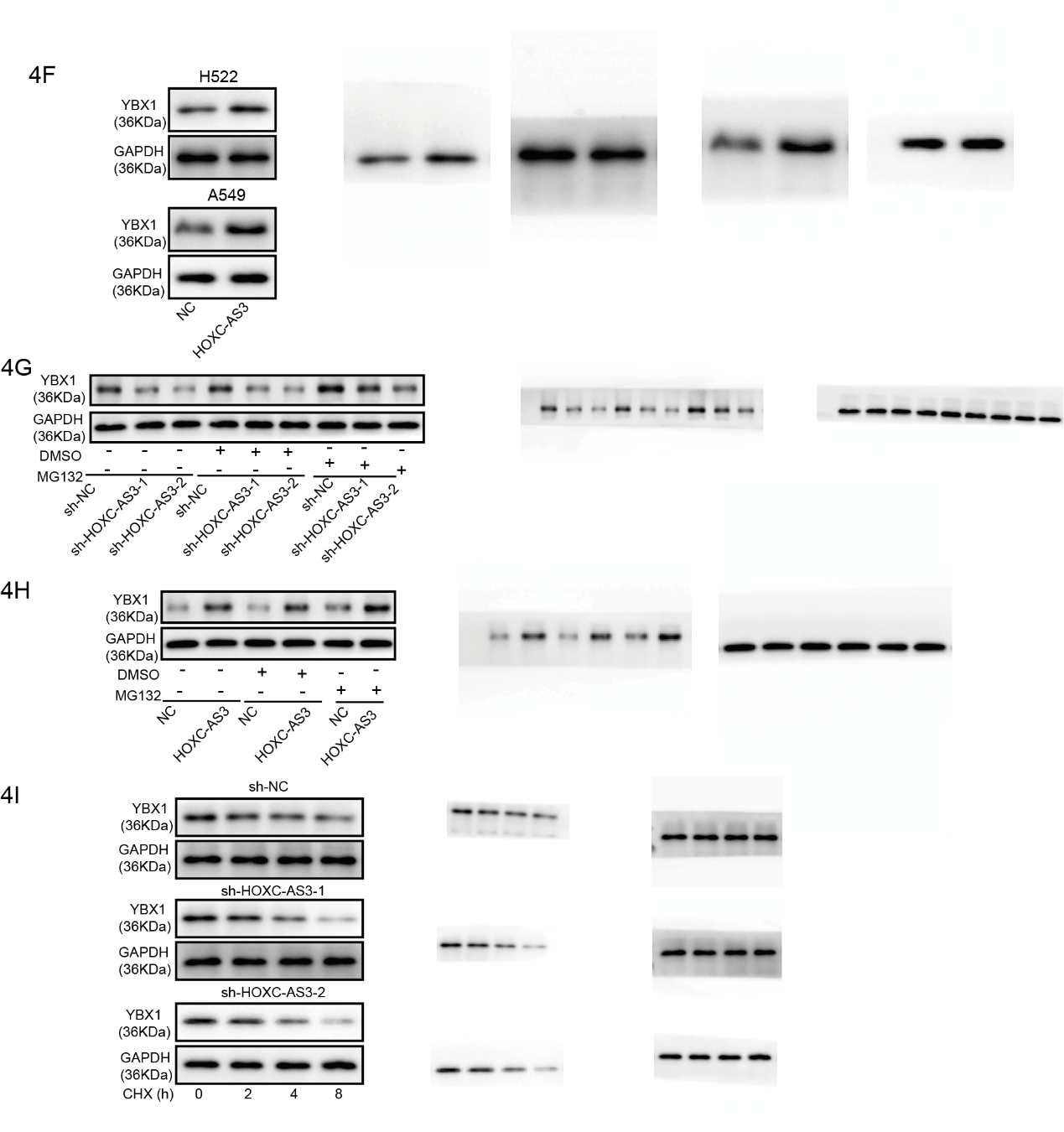

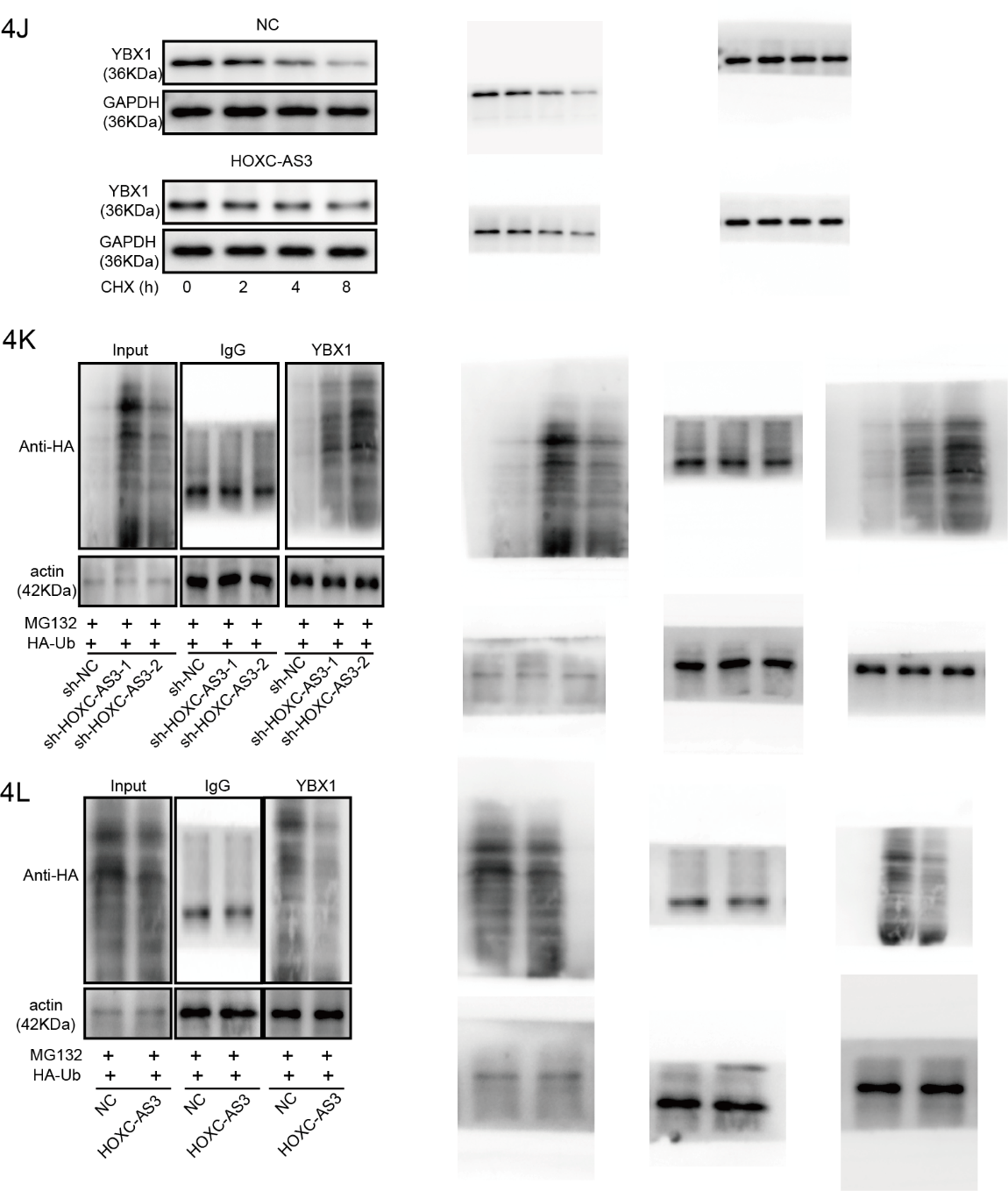

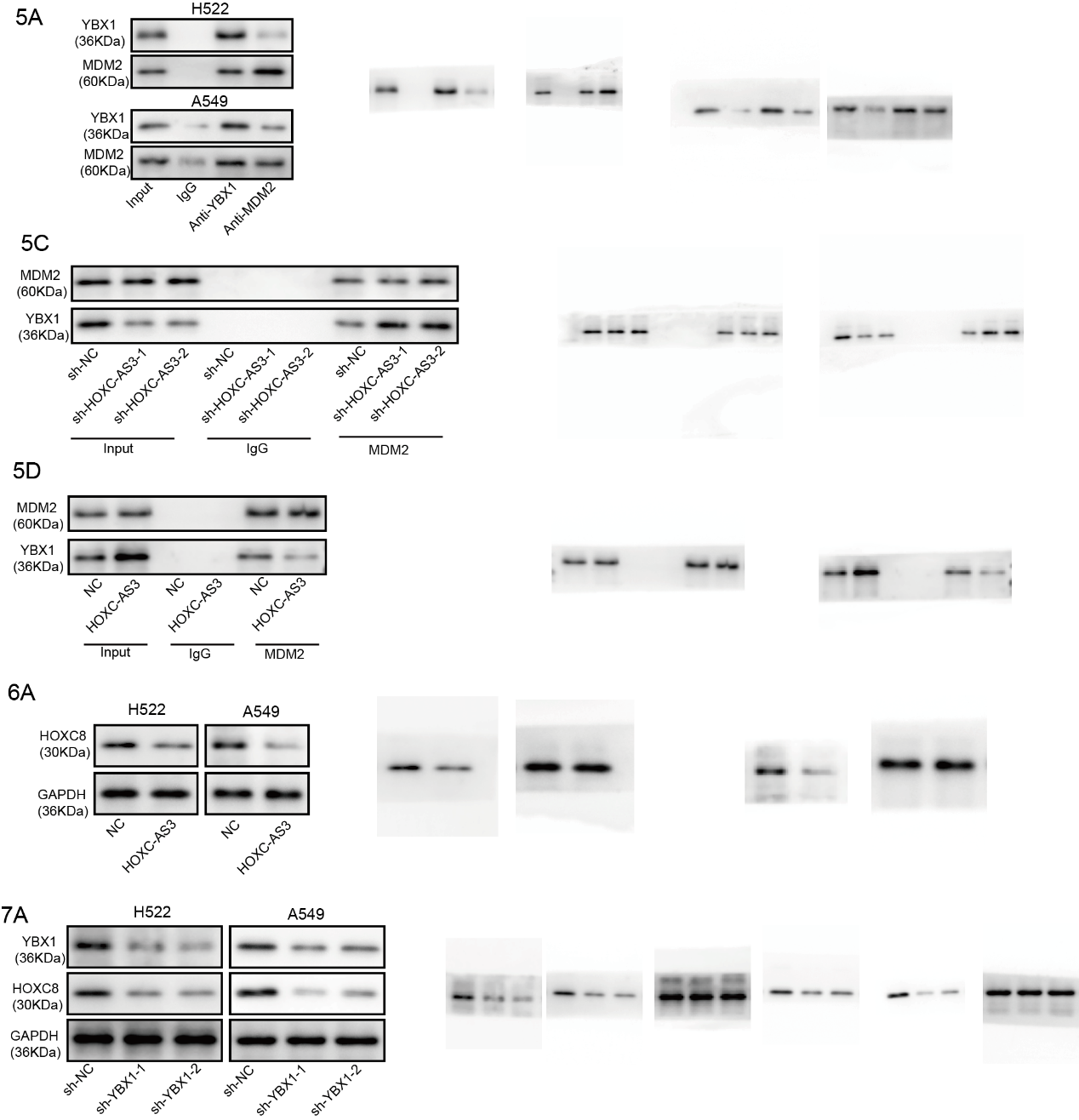

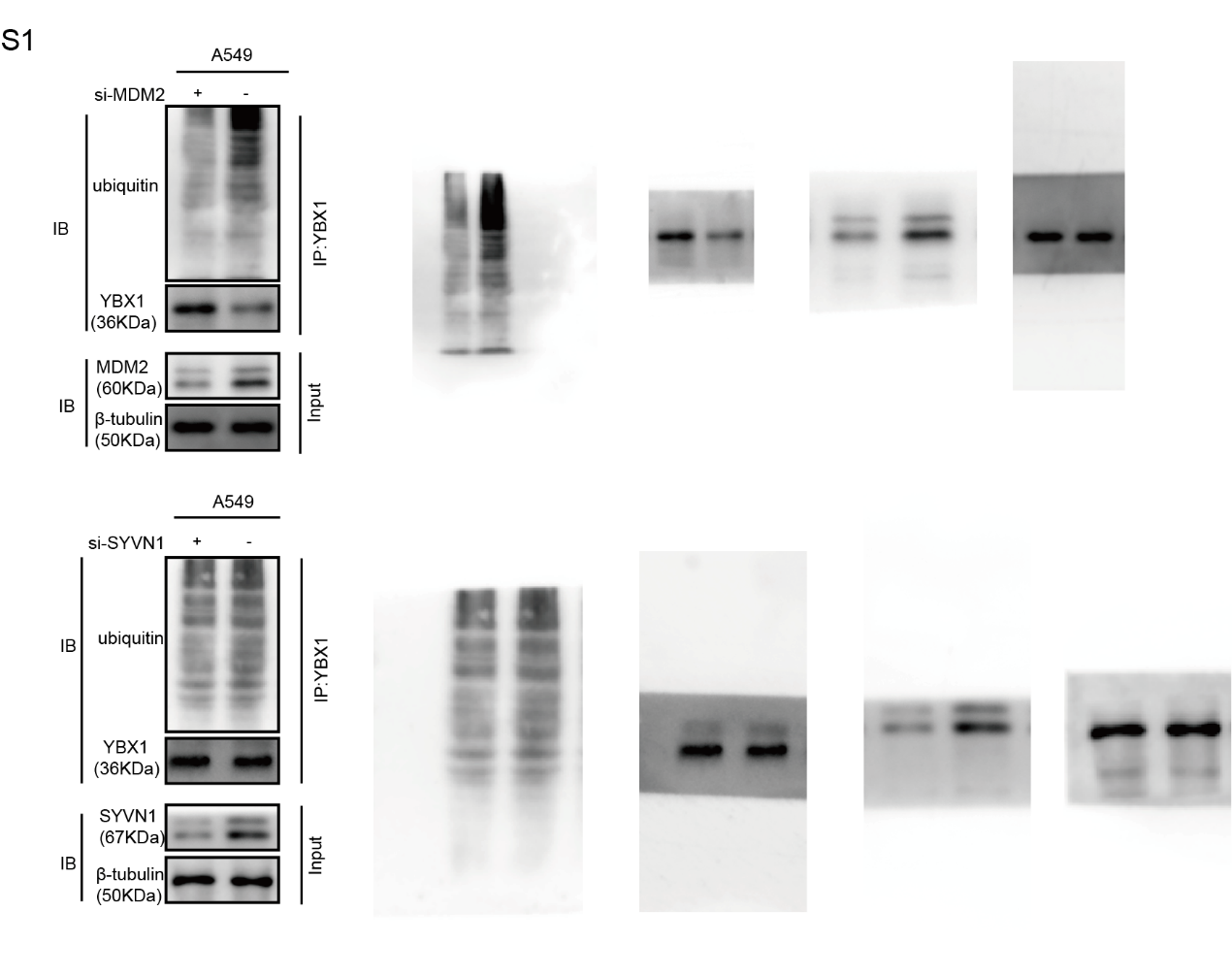

Supplement: Supplementary file 2 — WB [file 41419_2022_4723_MOESM2_ESM.docx]
